# Developing an implementation fidelity checklist for a vocational rehabilitation intervention

**DOI:** 10.1186/s40814-022-01194-x

**Published:** 2022-11-02

**Authors:** Katie Powers, Sara Clarke, Julie Phillips, Jain A. Holmes, Rachel Cripps, Kristelle Craven, Amanda Farrin, Roshan das Nair, Kathryn A. Radford

**Affiliations:** 1 Centre for Rehabilitation and Ageing Research, Queen’s Medical Centre, University of Nottingham, Nottingham, UK; 2 School of Population Health and Environmental Sciences, King’s College London, London, UK; 3 Clinical Trials Research Unit (CTRU), Leeds Institute of Clinical Trials Research, University of Leeds, Leeds, UK; 4 Mental Health & Clinical Neurosciences, University of Nottingham, Nottingham, UK; 5 Health Division, SINTEF, Trondheim, Norway

**Keywords:** Treatment fidelity, Measurement Development, Vocational rehabilitation, Occupational therapy, Complex interventions

## Abstract

**Background:**

Despite growing numbers of studies reporting the efficacy of complex interventions and their implementation, many studies fail to report information on implementation *fidelity* or describe how fidelity measures used within the study were developed. This study aimed to develop a fidelity checklist for measuring the implementation fidelity of an early, stroke-specialist vocational rehabilitation intervention (ESSVR) in the RETAKE trial.

**Methods:**

To develop the fidelity measure, previous checklists were reviewed to inform the assessment structure, and core intervention components were extracted from intervention descriptions into a checklist, which was ratified by eight experts in fidelity measurement and complex interventions. Guidance notes were generated to assist with checklist completion. To test the measure, two researchers independently applied the checklist to fifteen stroke survivor intervention case notes using retrospective observational case review. The scoring was assessed for interrater reliability.

**Results:**

A fidelity checklist containing 21 core components and 6 desirable components across 4 stages of intervention delivery was developed with corresponding guidance notes. Interrater reliability of each checklist item ranged from moderate to perfect (Cohen’s kappa 0.69–1).

**Conclusions:**

The resulting checklist to assess implementation fidelity is fit for assessing the delivery of vocational rehabilitation for stroke survivors using retrospective observational case review. The checklist proved its utility as a measure of fidelity and may be used to inform the design of future implementation strategies.

**Trial registration:**

ISRCTN, ISRCTN12464275. Registered on 13 March 2018.

**Supplementary Information:**

The online version contains supplementary material available at 10.1186/s40814-022-01194-x.

## Background

Poorly implemented interventions threaten participant and trial outcomes and undermine confidence in research findings. In intervention studies, it can be difficult to know whether interventions have been delivered as intended; that is, with fidelity [1, 2]. However, despite the body of literature supporting the importance of fidelity, it is largely under-reported in studies of rehabilitation interventions [3–5]. Without information regarding the extent to which an intervention has been delivered with fidelity, it is difficult to know whether the treatment effect outcomes are masked by poor implementation of the intervention [6]. Fidelity data are necessary to interpret intervention outcomes [1]. This is especially true of interventions with many interacting parts that are influenced by different contexts and factors, also called ‘complex’ interventions [7].

Complex interventions usually contain several ‘core components’ that are essential for the intervention to have an effect and to be considered as delivered with fidelity [8, 9]. The higher the level of complexity and individual tailoring of the intervention and its components, the more difficult it may be to measure fidelity [3, 10, 11], ,thus requiring a more sophisticated method of measurement to avoid drawing inappropriate conclusions about an intervention that might have been improperly implemented and making type III errors [12]. Measuring fidelity provides insight into which components of an intervention are essential for a positive participant outcome [13] by establishing what key components were or were not delivered in cases of improved outcomes [14].

Fidelity measurement is underpinned by theoretical concepts that emanate from behaviour change theories [1, 15, 16]. Various frameworks have been developed to describe and define which aspects of intervention implementation should be considered and the methods to use when evaluating fidelity [8, 15, 17, 18]. One such framework is the Conceptual Framework for Implementation Fidelity (CFIF) [17], which describes two key concepts to understanding implementation fidelity: (1) adherence (whether the recipient has received the intervention as intended) and (2) moderating factors (factors affecting faithful intervention implementation). Due to the comprehensiveness of this framework and its demonstrated usefulness in other complex intervention studies [18, 19], CFIF was used to define and describe fidelity in this study.

Some studies use quantitative data collection methods to measure elements of fidelity, such as fidelity checklists, that assess therapist adherence to core processes and determine which core intervention components have been delivered [5, 20]. Others use qualitative data collection methods, such as interviews, capturing acceptability, or engagement with the intervention experienced by participants [11]. These studies often do not include sufficient information regarding either the development of the fidelity measure used or the psychometric properties of the measure, which invites scepticism [5, 21–23]. The lack of published studies detailing the development of fidelity measures emphasises the need for future research to make clear the processes used to assure good psychometric properties of the measure prior to its application.

There is a need for high-quality, psychometrically robust measures of fidelity, yet there is little agreement on how best to develop these measures [5, 23]. Recent guidance suggests that for a measure to be considered high quality, the psychometric (e.g., reliability and validity) and implementation properties (practicality) of the measure should be reported [23]. Evaluation of a measure’s psychometric properties can determine whether the scores consistently measure the intended constructs [3, 24]. The practicality of a fidelity measure, such as ease of use and time taken to complete, is also valuable for researchers to report as these are factors that other researchers and clinicians consider when choosing a measure [3, 25].

Fidelity checklists are developed by using instructional information (i.e., intervention manuals), which is then distilled into a shortened list of intervention components and used to assess the presence of the components during delivery [23, 26]. Checklists have the advantage of being simple and quick to administer by those without specialist training in the intervention itself, and in instances where study participants cannot be, or do not wish to be, recorded or interviewed [5]. Assessment of fidelity through video or audio recordings of intervention delivery is currently considered the gold standard of assessment [27], but is resource intensive, especially in studies with many participants receiving intervention over an extended time period [23]. The application of a fidelity checklist using a retrospective review of intervention records might be a way to reduce resource use. Fidelity checklists have been generated in occupational therapy [3, 28]; however, they are specific to the components of the various interventions they assess and inappropriate for use across studies of other interventions without adaptation [3, 13].

Vocational rehabilitation (VR) is an example of a complex intervention that helps someone with a health problem return to or remain in work [29]. VR involves helping people find work, helping those who are in work experiencing difficulties, and supporting career progression in spite of illness or disability [30]. VR is complex because it requires tailoring of the intervention to the individual receiving it, is sensitive to the behaviours of different stakeholders, and can produce a variety of different outcomes [13]. VR crosses organisational boundaries, involves interactions between multiple stakeholders, is highly individually tailored, and requires behavioural change by the patient, their family, and employer [31, 32]. Stroke is an example of a particularly complex condition because it often occurs with multiple comorbidities and results in numerous, unpredictable biopsychosocial impacts [33]. Delivering a particularly complex intervention (such as VR) in a complex patient group (such as stroke survivors) presents some challenges for intervention delivery and measurement of fidelity (such as tailoring and individualization) to meet the specific needs of the recipients [34–36]. A small number of studies describe VR for stroke survivors [37], but very few of these studies report whether VR was delivered with fidelity, which makes it difficult to draw firm conclusions about the effectiveness of VR after stroke [38] despite the existence of intervention non-specific fidelity measures [8, 39].

**Fig. 1:**
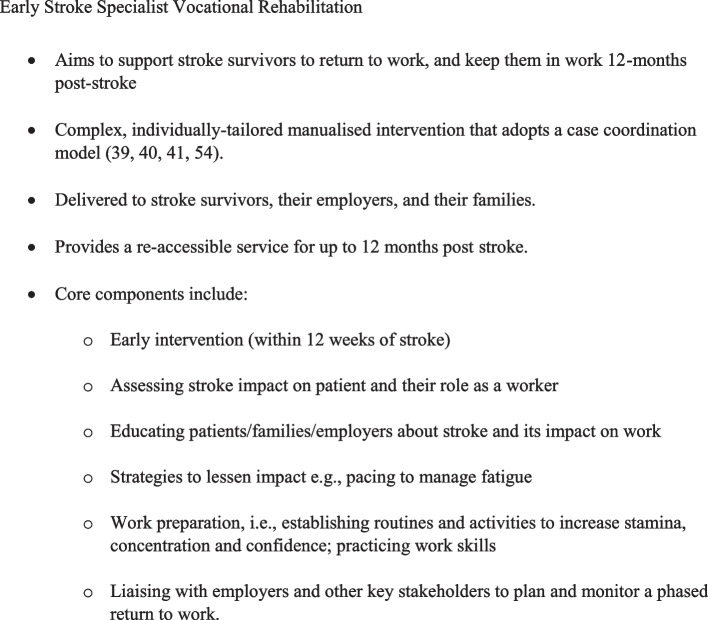
A brief description of ESSVR

This study describes the development and testing of an intervention fidelity checklist for an early, stroke specialist vocational rehabilitation intervention (ESSVR) to support stroke survivors to return to work after stroke in the REurn To work After stroKE (RETAKE) trial [40] (ISRCTN12464275). ESSVR combines conventional VR with case management (see Fig. 1). It is delivered by a stroke-specialist occupational therapist (OT) trained to assess the impact of the stroke on the participant and their job; coordinate appropriate support from the UK National Health Service (NHS), employers and other stakeholders; negotiate workplace adjustments, monitor return to work, and explore alternatives where current work is not feasible. A more detailed description of the intervention can be found elsewhere [41]. ESSVR is delivered in four stages (early recovery, graded return to work, job retention, and discharge), each comprising several core and desirable components.

### Aims and objectives

This study aims to develop and test a checklist for measuring implementation fidelity of ESSVR delivery in RETAKE.

Objectives:

1. To identify and extract core ESSVR intervention components and generate guidance notes to assess the fidelity of their delivery within the RETAKE trial.
2. To ratify the checklist components and guidance notes against expert opinion, supporting the measure’s content validity.
3. To test the utility of the checklist for assessing fidelity of ESSVR delivery using a retrospective observational review of stroke survivors’ intervention records.
4. To assess interrater reliability in fidelity checklist completion.

## Materials and methods

Ethical approval for the RETAKE trial and the studies within the trial was obtained through the East Midlands—Nottingham 2 Research Ethics Committee (REC) (Ref: 18/EM/0019).

### Development of the fidelity checklist

The development of the fidelity checklist and its associated guidance notes was informed by Walton et al. [23] and distilled into five steps: (1) review previous measures of fidelity, (2) analyse and develop a framework for the content of the intervention, (3) develop a fidelity checklist and associated guidance for checklist completion, (4) obtain feedback regarding content and wording, and (5) pilot and refine the checklist.

The initial structure of the ESSVR fidelity checklist was based on a checklist developed for an earlier VR study [42] for people with traumatic brain injury. The logic model and intervention descriptions [43] provided the initial content for the development of the ESSVR fidelity checklist.

The fidelity assessment in RETAKE used an observational retrospective review of stroke survivor ESSVR intervention records that included session content case-report forms (CRFs), OT clinical notes, and correspondence between the OT, stroke survivor, and other key stakeholders to assess intervention fidelity (see Table 1). ESSVR was delivered to community-dwelling stroke survivors, their families, and their employers over a period of up to 12 months following randomisation.

**Table 1:** Detailed descriptions of the components of the participant ESSVR intervention records

Intervention record component	Description
Content CRFs	Details each intervention session. OTs assign 10-min units to components of the intervention and other common OT practices.
OT clinical notes	OT notes from each point of contact with the stroke survivor and key stakeholders.
Supplementary material	Extra materials provided in the case file. Includes: 1. Evidence of correspondence (e.g., copies of emails and written communication to key stakeholders) 2. Educational information provided to key stakeholders.

### Version 1

Version 1 of the checklist used the same format as a fidelity checklist created to assess a similar VR intervention delivered to people with traumatic brain injury [42] designed to be completed through observation of individual sessions. Both the VR intervention designed for people with traumatic brain injury and the VR intervention for stroke survivors require complex, highly individualised intervention that considers the patient’s individual, family, and work contexts. The VR in TBI checklist was developed to be completed through direct observation of a therapy session whereby the assessor recorded the extent of delivery (‘always’, ‘sometimes’, ‘seldom’, or ‘never’ delivered) for each of the 18 components of the intervention in the session. The assessor was also prompted to record moderating factors impacting intervention delivery or receipt, such as participant responsiveness and political, economical, and organisational context [42].

The checklist used for VR following traumatic brain injury was adapted for use in this study by modifying existing components and adding additional components identified in the ESSVR logic model. The process to complete the checklist was adapted to use observation of stroke survivor intervention records to assess the delivery of intervention components across the entire intervention delivery period (up to 12 months). It required the fidelity assessors (KP, RC), who were research assistants with a background in psychology and no training in OT or VR, to determine the frequency with which a component was delivered by the RETAKE OT (‘always’, ‘sometimes’, ‘seldom’, or ‘never’ delivered) and included a space for the assessor to record moderating factors that may have facilitated or prevented faithful delivery or receipt of each component. There were no accompanying guidance notes to aid interpretation or completion.

### Piloting of version 1 and proposed changes

The research assistants (KP, RC) applied version 1 of the fidelity checklist to 8 sets of participant intervention records collected from the ESSVR *feasibility study* [41]. The intervention records were first read for familiarisation before data were extracted against the checklist components. Following piloting, changes were made to increase clarity and facilitate administration (see Table 2).

**Table 2:** Description of changes from previous versions

Fidelity checklist version number	Changes made from previous version
Version 1	Amalgamated RETAKE logic model content and the FRESH fidelity checklist structure to create a physical checklist of the ESSVR intervention components.
Version 2	Assessor no longer asked about the frequency with which the OTs delivered the intervention. Assessor asked about the evidence of the extent to which each component of the intervention: ‘no evidence’, ‘some evidence’ and ‘extensive evidence’. Requires evidence of component delivery to be extracted verbatim from participant’s case file.
Version 3	Assessor no longer asked to evaluate the extent of the evidence of the component and instead asked to decide whether there is ‘adequate’ evidence of the component with dichotomous answers ‘adequate evidence’ and ‘not adequate evidence’. Components added from RETAKE ESSVR training manual. Components separated into intervention stages (early recovery and work planning, graded return to work, job retention and discharge process). Guidance notes were developed for use alongside the checklist.
Version 4	Every item on the checklist is answered with ‘Yes’, ‘No’ or ‘Not applicable’ Components are categorised as either ‘core’ or ‘desirable’ Components delivered to ‘participant, participant’s family, and participant’s employer’ made separate and addressed individually. Includes a box for the assessor to record the amount of time taken to complete the checklist.
Version 5	The checklist changed from Word to Excel Spreadsheet containing formulae to automatically sum core and desirable components within and across intervention stages. The formula is written to calculate the 12-week cut-off date for the point of first contact with OT (Core Component 1) Drop drown menus added for ‘Evidence Source’ for use when adding evidence from the case file. Data is then extracted into the next cell. Drop down menus for Yes/No/Not deliverable when completing the checklist.

These proposed changes were discussed by members of the research team comprising an experienced stroke and OT researcher (KR), research OTs with experience in designing and implementing fidelity checklists (JH, JP), and research assistants with no clinical background who developed and implemented the fidelity checklist in this study (KP, RC, SC). Agreed changes were incorporated into a new version of the fidelity checklist (version 2).

### Piloting of version 2 and production of guidance notes

The revised checklist was independently piloted against a further 10 sets of participant intervention records from the feasibility trial by two research assistants (KP, RC) who met to discuss discrepancies in administration and data extraction. Two clinical-academic OTs familiar with the intervention and responsible for training therapists in its delivery were consulted where there were discrepancies or questions regarding the intervention components. The ESSVR manual was also consulted for clarification. The piloting and consultation led to the development of version 3 of the checklist. Guidance notes for checklist administration were developed with reference to the intervention training manual and with input from the RETAKE OT training team.

The guidance notes explain each component of the intervention in detail, providing definitions of key phases and concepts to assist the person administrating the checklist. The guidance notes also give examples of where to find the evidence to support each component.

### Expert panel

An expert panel was then formed to foster opinion from researchers with a clinical background and/or fidelity measurement expertise in relation to complex rehabilitation trials.

The expert panel consisted of eight researchers with both expertise in fidelity measurement and experience in measuring fidelity in complex rehabilitation trials. The purpose of the expert panel was to assist in (1) distinguishing between the ‘core’ and ‘desirable’ components of the intervention, (2) defining keywords and phrases within the fidelity checklist and guidance notes, and (3) assessing the suitability of the fidelity checklist and accompanying guidance notes.

Version 3 of the fidelity checklist and version 1 of the guidance notes were emailed to the expert panel members prior to the meeting. During the meeting, KP presented an anonymized participant intervention record from the feasibility study to the expert panel. The participant’s case was used to illustrate the application of the fidelity checklist and promote discussion of the components.

The panel discussed the core and desirable components of the intervention, practical application of the fidelity checklist, and the potential limitations of the methodology (e.g., method relies on OT record keeping), providing feedback and suggestions for amendments.

The feedback resulted in version 4 of the fidelity checklist and version 2 of the guidance notes.

### Piloting of versions 4 and 5 of the fidelity checklist and version 2 of the guidance notes

Version 4 was independently piloted by two research assistants (KP, SC) on a further two cases from the RETAKE trial and the discrepancies discussed. No changes were made to the fidelity checklist and only minor changes were made to the guidance notes where further clarification was needed.

A digitised version of the checklist was created in Microsoft Excel and piloted by a third researcher with no clinical background, with no prior involvement in the fidelity checklist development to test the functionality of the digitised checklist. No further changes were made.

### Interrater reliability

Participant intervention records for 15 ESSVR recipients were selected at random to assess interrater reliability. Treating OTs were asked to redact identifiable information and upload the anonymized intervention records to a secure file transfer service. Two independent researchers (KP and JP), one with no background in OT or VR (KP), and one expert in VR and OT who was instrumental in the development of the intervention (JP), independently applied the fidelity checklist assisted by the guidance notes.

A Cohen’s kappa statistic was calculated to assess interrater reliability. Based on guidelines for the interpretation of Kappa values, a value between 0 and 0.20 indicates no to slight agreement, 0.21 and 0.39 minimal agreement, 0.40 and 0.59 weak agreement, 0.60 and 0.79 moderate agreement, 0.80 and 0.90 strong agreement and 0.90 and above almost perfect agreement [44].

## Results

### Development of fidelity checklist and guidance notes

Two materials were produced to aid in the assessment of fidelity in RETAKE: the fidelity checklist and its accompanying guidance notes (see Supplementary Files). The fidelity checklist was structured into the four stages of the intervention as described in the OTs’ intervention manual: early recovery, graded return to work, job retention, and discharge process.

To implement the checklist, the fidelity assessor was asked to review each participant’s intervention record. For each component, the assessor was asked whether there was sufficient evidence of the component’s delivery, where the assessor could select ‘Yes’, ‘No’, or ‘Not deliverable’ from a drop-down menu. The checklist provided space for the assessor to record details verbatim from the intervention record that would either evidence where the component had been delivered or provide evidence for why the component was not deliverable (moderating factors; e.g., where the OT did not have consent to contact an employer).

### Piloting of version 1 and proposed changes

Across Versions 1–3 of the fidelity checklist, changes were made to the structure and content to best capture the core components of the intervention, increase clarity, and facilitate the administration of the checklist. Version 1 listed 10 core components. Proposed changes related to the evaluation of component delivery where ‘frequency’ was replaced with ‘no evidence’, ‘some evidence’, and ‘extensive evidence’, and a box was created to extract the supporting evidence verbatim into the checklist.

For full description of changes made to each version of the checklist, see Table 2.

### Piloting of version 2 and production of guidance notes

During the piloting of version 2, the OT training manual was consulted. This provided the biggest structural difference in the checklist. Consulting the training manual resulted in the classification of intervention components into four phases (1, early recovery; 2, graded return to work; 3, job retention; and 4, discharge process) to mirror the information provided to the RETAKE OTs. Additional components specific to work monitoring and discharge processes were extracted from the RETAKE OT training manual. These components were highlighted as being essential to intervention delivery but were not explicitly listed in the logic model.

### Expert panel

Version 3 of the checklist and version 1 of the guidance notes were taken to the expert panel. The expert panel facilitated discussion regarding the core components and their status as ‘core’ or ‘desirable’ to the intervention delivery. Based on feedback from the expert panel, the components and other key concepts and phrases were more clearly defined in the guidance notes. Jargon was minimised to improve the clarity and accessibility of the guidance notes.

The expert panel agreed that in addition to evidencing each component verbatim from the intervention records, the assessors should record the source of the evidence (e.g., correspondence, therapy notes). The expert panel also agreed that the assessor should record how long it takes to complete each fidelity assessment to evaluate the speed of checklist completion and compare it to other methods of fidelity assessment.

### Versions 4 and 5

Version 4 of the fidelity checklist and version 2 of the guidance notes were produced which incorporated the recommendations from the expert panel. Following the application of the checklist to two further sets of ESSVR participant intervention records, the fidelity checklist was digitised into a Microsoft Excel spreadsheet to increase its utility. The spreadsheet contained a drop-down menu for the assessor to select whether there was sufficient evidence of the component or if the component was not deliverable. The assessor was then directed to provide evidence verbatim from the intervention record where possible in the next box where the assessor was also asked to select the source (CRF, clinical case notes, correspondence, etc.) from another drop-down menu.

Scoring of the fidelity checklist was written into a calculation which was automatically populated via the drop-down menu selection of ‘Yes’, ‘No’, and ‘Not deliverable’. The total overall fidelity score was calculated based on the number of delivered components divided by the number of components that were deliverable. Components that were classified as ‘desirable’ were only included in the calculation where they were delivered and were thus weighted differently than those classified as ‘core’, e.g.,

$$\left(\frac{n\ core\ components\ delivered+n\ desirable\ componets\ delivered}{N\ core\ components-n\ undelivereable\ core\ components+n\ desireable\ components\ delivered}\right)\times 100=\%\textrm{fidelity}$$

### Interrater reliability

Assessment of 15 participant intervention records was completed by two independent assessors. The stroke survivors whose records were used to assess interrater reliability included six females (40%) and ages ranged from 33 to 61 years old (mean: 48.3 years, SD: 7.7). Cohen’s kappa ranged from 0.69 to 1 (See Table 3). Eleven items achieved 100% agreement, eight items achieved 90% agreement, and eight items achieved 80% agreement.

**Table 3:** Assessment of interrater reliability per checklist item

Stage	Item	Component description	Core or desirable	Cohen’s Kappa	95% CIs
1. Early recovery	1.1	OT intervenes within 12 weeks of stroke.	Core	1	1
	1.2	OT assesses the impact of the stroke on the participant.	Core	a	
	1.3	OT assesses the impact of the stroke on the participant’s job.	Core	a	
	1.4	OT assesses the impact of the stroke on the participant’s family.	Desirable	0.79	0.56–0.89
	1.5	OT helps participant plan a return to work and prepares them to return to work.	Core	1	1
	1.6	OT communicates in writing with relevant stakeholders regarding work status.	Core	a	
	1.7	OT coordinates VR across relevant sectors.	Core	0.74	0.44–0.87
2. Graded return to work	2.1	OT provides education and advice to the participant.	Core	1	1
	2.2	OT provides emotional support to the participant.	Desirable	0.83	0.73–1.0
	2.3	OT provides education and advice to the employer.	Core	0.82	0.61–1.0
	2.4	OT provides emotional support to the employer.	Desirable	0.76	0.73–1.0
	2.5	OT provides education and advice to the participant’s family.	Desirable	0.79	0.69–1.0
	2.6	OT provides emotional support to the participant’s family.	Desirable	0.79	0.69–0.88
	2.7	OT negotiates a phased return to work.	Core	1	1
	2.8	OT mediates workplace adjustments.	Core	1	1
	2.9	OT provides a mechanism for feedback based on work performance.	Core	0.79	0.66–0.89
	2.10	OT continuously monitors the participant’s return to work to ensure sustainability and job retention.	Core	1	1
3. Job retention	3.1	OT identifies issues that arise within the return-to-work process with relevant stakeholders.	Core	0.75	0.58–0.89
	3.2	OT addresses issues that arise within the return-to-work process with all stakeholders.	Core	0.69	0.61–0.78
	3.3	OT explores alternative duties and/or job roles with the participant where current work could not be sustained/ was not feasible.	Core	0.76	0.63–1.0
	3.4	OT practices gradual, appropriate disengagement from intervention with the participant.	Core	1	1
	3.5	OT discusses gradual, appropriate disengagement from intervention with the participant’s employer.	Core	0.79	0.48–0.89
4. Discharge	4.1	OT and participant agree on an appropriate timepoint for withdrawing from intervention.	Core	0.81	0.69–1.0
	4.2	OT discusses and communicates mechanisms for re-accessing vocational services or provides information about access to further avenues of support to the participant.	Core	0.87	0.62–1.0
	4.3	OT discusses and communicates mechanisms for re-accessing vocational services or provides information about access to further avenues of support to the participant’s employer.	Core	0.9	0.79–1.0
	4.4	OT discusses and communicates mechanisms for re-accessing vocational services or provides information about access to further avenues of support to the participant’s family.	Desirable	0.77	0.55–0.89
	4.5	OT provides the participant’s GP and other relevant health care professionals with a copy of the discharge letter.	Core	1	1

^a^Both researchers rated this item as having sufficient evidence in every case. The lack of variation in scoring made it impossible to calculate a Cohen’s kappa

### Time taken to complete

The time taken to complete the fidelity checklist ranged from 30 to 100 min (average 62 min). The average time taken to complete per assessor was 63.5 min (KP) and 57 min (JP).

## Discussion

An ESSVR-specific fidelity checklist with adequate interrater reliability, that is relatively quick to apply, and guidance notes to aid checklist completion were developed and piloted using the observational retrospective review of ESSVR participant intervention records. The checklist is adaptable to the specific contexts of the stroke survivors and other stakeholders and captures factors affecting the delivery of each component and facilitating identification and categorisation of implementation considerations. A future study will evaluate and report the fidelity of ESSVR delivery and factors affecting the delivery of individual components in RETAKE.

Application of the fidelity checklist to assess interrater reliability produced a Cohen’s kappa score ranging from 0.69 to 1, which indicates moderate to perfect interrater reliability [44]. Previous studies of fidelity checklist development report difficulties in obtaining high levels of agreement [2]. It is possible that this study achieved higher agreement through the information provided to the fidelity assessors through the guidance notes. It is also possible that this could be due to the involvement of the assessors with the ESSVR training team, which may have influenced the interpretation of the data in the ESSVR participant intervention records. Further research should explore whether other assessors with differing backgrounds would obtain the same high level of agreement. This study assessed interrater reliability using 15 stroke survivors’ intervention case notes, which is a small sample, but the results lend valuable information regarding how to improve the guidance notes to aid further understanding and agreement.

Of the eleven items within the checklist that yielded ‘moderate’ agreement, six of the items were core components and five were desirable components. The desirable components that produced ‘moderate’ agreement related to the OT’s delivery of an ESSVR component to the stroke survivor’s family or the delivery of emotional support to the employer. In exploring this further, the researchers completing the checklist disagreed on whether these components were deliverable or not as opposed to the presence of sufficient evidence. An example of where these components would not be deliverable is if a stroke survivor expressed, explicitly or implicitly, that they would prefer their family not be involved in their intervention. Future applications of the checklist should take this into account and guidance notes should be altered to provide further clarity. Of the six core items, three items asked the researchers to determine the delivery of a component to relevant ‘stakeholders’ or ‘sectors’. It is possible that the disagreement on these items was related to a lack of sufficient clarity in the guidance notes around the range of specific relevant stakeholders this might refer to. The other three core components that produced ‘moderate’ agreement all involved OT communication with the participant’s employer. The delivery of these components was impacted by factors outside of the OT’s control (e.g., employer engagement with the OT), which may explain discrepancies in the raters’ marking. Updates to the guidance notes to reflect this and support future applications are warranted.

Consultation with the expert panel provided a way to evaluate and establish the checklist’s content validity. Expert ratification of a measure’s components and scoring is a common way to evaluate content validity and confirm that the measure is assessing what it intends to assess [45, 46]. Recommendations for what constitutes as a suitable expert panel to establish content validity suggest that the members should be professionals with experience in the subject matter or clinical/research experience in the field [47]. This study’s expert panel comprised eight researchers with expertise in fidelity measurement within studies of complex interventions. Two of the researchers also had extensive knowledge and clinical experience of occupational therapy, vocational rehabilitation and ESSVR itself. By adopting the recommendations of the expert panel and adapting the checklist and its guidance notes, content validity was established. This study did not use a measure to quantify content validity, but this should be considered in future research to strengthen the measure [48]. Additionally, the expert panel did not include a representative from the trial’s Patient and Public Involvement group which would have provided added benefit in understanding what intervention components were of greater importance to those receiving it.

The time taken to apply the checklist ranged from 30 to 100 min. For context, a typical ESSVR session with a stroke survivor might be expected to last 30 to 60 min and a stroke survivor might expect have over a dozen sessions over the course of 12 months in some cases. The variation in time taken to complete the checklist was most likely due to the variation in the amount of information included within each ESSVR participant intervention record. Fidelity measurement research highlights the practicality of the measure (i.e., quick and easy use) as helpful for conserving resources [49] and reducing the burden within a study [5, 50]. The time to complete the measure in this study using observational retrospective case review provides a considerably quicker method to assessing an entire period of intervention delivery when compared with studies using more direct observational methods [5, 51].

The associated guidance notes facilitated the checklist’s use and provided a way to support the application of the checklist without having to provide additional training for future assessors. In the earlier stages of the checklist development, the research assistants initially applying the checklist frequently met with the research OTs responsible for training the RETAKE OTs to discuss discrepancies in the interpretation and adequate demonstrations of component delivery, which aided the development of the guidance notes. These were refined to thoroughly cultivate understanding and aid practicality, which might further explain the adequate level of agreement and interrater reliability between the raters (KP & JP). The thorough process used to create and refine the guidance notes facilitated ease of checklist administration, which is another important aspect of measure implementation that studies of fidelity measures often fail to report [5, 21]. With clear guidance notes facilitating sufficient levels of agreement, even where the person applying the checklist does not have a clinical background or experience in the intervention delivery, valuable study resources (e.g., clinical staff capacity and costings) may be conserved and may reduce bias. However, the results of the interrater reliability assessment are limited by the lack of a sensitivity analysis to determine what factors might have further influenced interrater reliability.

The intention of this study was to develop a checklist that could be applied by a research assistant in a trial, thereby reducing the risk of bias. Arguably, if the checklist is robust and guidance notes clear and the OTs adequately document the intervention, then a non-clinician should be able to extract the data and apply the checklist, saving valuable clinical and research study resources, particularly given the high costs and capacity issues associated with the use of clinical staff. This approach is in no way intended to devalue clinical experience or expertise in the delivery of this or any other complex interventions, but rather aims to provide an efficient way of measuring fidelity during a clinical trial. Experienced clinical mentors overseeing the clinical implementation of the intervention [52] could be informed of deviations from the process and address these in real time during the trial, further facilitating faithful delivery of the intervention.

There are some limitations to this method of assessing fidelity. Using the observational retrospective review of ESSVR participant intervention records in this study meant that fidelity checklist completion was dependent on the detailed record keeping of the RETAKE OTs. This limited the conclusions to whether there was sufficient evidence of the component’s delivery. In cases where there was not sufficient evidence of a component’s delivery, we could not confidently conclude that it had not been delivered. Direct observation of intervention delivery either in-person or via audio/video-recorded sessions is an effective way of confidently determining whether or not an intervention component has been delivered [23, 27]. However, whilst observation of intervention delivery is an established rigorous approach to assessing fidelity, it is not always possible or feasible as participants might not always give consent for session recording [5] and this approach also requires considerable staff and time resource [5, 53]. Direct observation of intervention delivery might also cause the person delivering the intervention to behave differently to when they are not being observed [26, 54, 55].

To help draw more confident conclusions about component delivery using observational retrospective case review, future research might include a method to further assist or encourage therapists in detailed record keeping through an electronic database, for example. Future implementation studies might also use this approach of assessing fidelity to support the faithful delivery of interventions whereby intervention records could be reviewed on an ongoing basis starting from the beginning of intervention delivery. This approach would enable researchers to identify intervention components that are not being consistently delivered and support those delivering the intervention to deliver these components in the future. Future research may also look to expand upon this method and the checklist, making it more robust by defining parameters for the *amount* of evidence present and assigning levels of sufficiency beyond ‘Yes’ ‘No’, and ‘Not deliverable’ with reference to the components. Lastly, future research should seek to involve clinicians in further development and testing of the checklist, where the rates of interrater reliability could then be compared with those of non-clinicians.

This approach to measuring fidelity allowed us to observe the intervention delivery over long periods of time over an unprescribed number of sessions across multiple study centres with multiple therapists. Whilst the checklist components are specific to the ESSVR intervention, the process followed to develop and apply the checklist is replicable and generalisable to studies of complex interventions. This approach may inform the design of implementation strategies in future studies of complex interventions.

## Conclusion

The checklist and guidance notes developed in this study are fit for assessing the delivery of ESSVR components in the RETAKE trial, and their application will be essential in providing context for the interpretation of the results of the trial with regard to the effectiveness of the intervention. The process followed to create the fidelity checklist in this study will inform the design of future implementation strategies for complex rehabilitation interventions.

This study also considered the feasibility of using a retrospective review of intervention records to assess fidelity, which may facilitate robust longitudinal fidelity assessment procedures in future complex intervention studies. Establishing robust methods of assessing fidelity in complex rehabilitation interventions, such as ESSVR, will help researchers more confidently draw conclusions about the effectiveness of the interventions they seek to evaluate.

## Supplementary Information

**Additional file 1.** ESSVR Fidelity Checklist. Digitised version of the ESSVR fidelity checklist developed in this study. ESSVR Fidelity Checklist Completion Guidance Notes. Guidance notes developed in this study to aid in checklist completion.

## Data Availability

On study completion, the final trial dataset will be archived at the University of Nottingham. Following completion of the RETAKE trial and publication of its effectiveness outcomes, any party may apply to the corresponding author for access to the dataset. Access will be governed by an information governance committee formed between the University of Nottingham and the University of Leeds.

## Abbreviations

CFIF: Conceptual Framework for Implementation Fidelity
CRF: Case-report form
ESSVR: Early, stroke-specialist vocational rehabilitation
NHS: National Health Service
OT: Occupational therapist
RETAKE: REturn To work After stroKE trial
VR: Vocational rehabilitation
