# Supplementary material for: Developing an implementation fidelity checklist for a vocational rehabilitation intervention
Source: Pilot Feasibility Stud. 2022 Nov 2;8:234. doi: 10.1186/s40814-022-01194-x (PMC9628165; doi:10.1186/s40814-022-01194-x)
Supplement: Supplementary file 1 — Additional file 1. ESSVR Fidelity Checklist. Digitised version of the ESSVR fidelity checklist developed in this study. ESSVR Fidelity Checklist Completion Guidance Notes. Guidance notes developed in this study to aid in checklist completion. [file 40814_2022_1194_MOESM1_ESM.zip › FidelityAssessmentGuidanceNotes ESSVRR1.docx]

Fidelity Assessment Guidance Notes

Table of Contents

[Stage One: Early Recovery and Work Preparation 3](#_Toc66709377)

[1.1 ESSVR OT intervenes within 12 weeks of stroke 3](#_Toc66709378)

[1.2 ESSVR OT assesses the impact of the stroke on the participant 3](#_Toc66709379)

[1.3 ESSVR OT assesses the impact of the stroke on the participant’s job 4](#_Toc66709380)

[1.4 ESSVR OT assesses the impact of the stroke on the participant’s family (desirable) 4](#_Toc66709381)

[1.5 ESSVR OT helps the participant plan a return to work and prepares them to return to work 5](#_Toc66709382)

[1.6 ESSVR OT communicates in writing with stakeholders regarding work status 7](#_Toc66709383)

[1.7 Coordinates VR across relevant sectors 8](#_Toc66709384)

[Stage Two: Graded Return to Work 9](#_Toc66709385)

[2.1 Provides education and advice to the participant 9](#_Toc66709386)

[2.2 ESSVR OT provides emotional support to the participant (desirable) 9](#_Toc66709387)

[2.3 Provides education and advice to the employer 10](#_Toc66709388)

[2.4 ESSVR OT provides emotional support to the employer (desirable) 10](#_Toc66709389)

[2.5 Provides education and advice to the participant’s family (desirable) 11](#_Toc66709390)

[2.6 ESSVR OT provides emotional support to the participant’s family (desirable) 11](#_Toc66709391)

[2.7 ESSVR OT negotiates a phased return to work 11](#_Toc66709392)

[2.8 ESSVR OT mediates workplace adjustments 12](#_Toc66709393)

[2.9 ESSVR OT provides mechanism for feedback based on work performance: 13](#_Toc66709394)

[2.10 ESSVR OT continuously monitors the participant’s return to work to ensure sustainability and job retention 13](#_Toc66709395)

[Stage Three: Job Retention 14](#_Toc66709396)

[3.1 ESSVR OT identifies issues that arise within the return-to-work process with relevant stakeholders 14](#_Toc66709397)

[3.2 ESSVR OT addresses the issues that arise within the return-to-work process with all stakeholders. 14](#_Toc66709398)

[3.3 ESSVR OT explores alternative duties and/or job roles for the participant where current work could not be sustained/ was not feasible 15](#_Toc66709399)

[3.4 ESSVR OT practices gradual, appropriate disengagement from the intervention with the participant 15](#_Toc66709400)

[3.5 ESSVR OT discusses gradual, appropriate disengagement from the intervention with the participant’s employer 16](#_Toc66709401)

[Stage Four: Discharge Process 16](#_Toc66709402)

[4.1 ESSVR OT and participant agree an appropriate timepoint for withdrawing from the intervention 17](#_Toc66709403)

[4.2 Discusses and communicates the mechanism for re-accessing the vocational service or provides information about access to further avenues of support to the participant 17](#_Toc66709404)

[4.3 Discusses and communicates the mechanism for re-accessing the vocational service or provides information about access to further avenues of support to the participant’s employer 18](#_Toc66709405)

[4.4 ESSVR OT discusses and communicates the mechanism for re-accessing the vocational service or provides information about access to further avenues of support to the participant/ participant’s employer/ participant’s family (desirable) 18](#_Toc66709406)

[4.5 ESSVR OT provides the participant’s GP and other relevant health care professionals with a copy of the Discharge Letter 18](#_Toc66709407)

[Other Modifications to the ESSVR Protocol 19](#_Toc66709408)

[Fidelity Checklist Completion 20](#_Toc66709409)

# Stage One: Early Recovery and Work Preparation

## 1.1 ESSVR OT intervenes within 12 weeks of stroke

For the intervention to have been considered to have been delivered ‘early’, the ESSVR OTs must have intervened within 12 weeks of the participant’s stroke. ‘Intervening’ can include the initial phone call to introduce themselves as the participant’s ESSVR OT. This first contact should be documented on the **Occupational Therapy ESSVR Assessment Form** and/or the **Therapy Case Notes**. Input the date of the participant’s stroke where indicated in the spreadsheet in dd/mm/yyyy format. The spreadsheet will calculate the date by which the participant will have needed to be seen by. Input the date of first contact as evidence. Select the source of evidence. Instances where participants were still in hospital should be considered as ‘No’ in most cases as many OTs are able to contact the participant in hospital. ‘Not Deliverable’ would be an appropriate selection if the participant is not engaging with the OT despite the OT’s attempts to contact the participant.

Select ‘Yes’ if ESSVR OT made contact with participant within 12 weeks of their stroke.

Select ‘No’ if ESSVR OT did not make contact with participant within 12 weeks of their stroke and record any reason that this was delayed.

Select ‘Undeliverable’ if ESSVR OT did not make contact with participant despite many evident attempts to do so.

1.2 ESSVR OT assesses the impact of the stroke on the participant

This could be evidenced/documented in the Occupational Therapy ESSVR Assessment Form and/or the Therapy Case Notes:

*Functional assessments: This will help the ESSVR OT determine what skills and areas have been affected by the stroke. ESSVR OTs have been encouraged to begin with physical skills as they are easier for the participant to identify. In addition to attempting to gain an overall picture of the impact of the stroke on the participant, these functional assessments should also attempt to assess skills that might be similar to work tasks (computer skills, telephone skills, emails, report writing, manual tasks at home). ESSVR OTs are encouraged to think about fatigue and impact on performance.*

*Standardised assessments: These should be conducted if they are required and available locally. In cases where standardised assessments have been or may have been conducted by another service, there should be evidence of liaison with the appropriate healthcare services (i.e., physiotherapy, neuropsychological, OT, and speech and language services).*

*Social impact: ESSVR OTs might assess whether the stroke has had an impact on the participant’s ability to cope with social interaction.*

Select ‘Yes’ if ESSVR OT assessed the impact of the stroke on the participant and extract evidence verbatim from case file.

Select ‘No’ if ESSVR OT did not assess the impact of the stroke on the participant and detail any reasons for why this was not completed.

Select ‘Not Deliverable’ if there is a reason perceived for not assessing the impact of the stroke and detail the reason.

## 1.3 ESSVR OT assesses the impact of the stroke on the participant’s job

Analysis of work ability and/or worksite assessment:

*The ESSVR OT should conduct a thorough assessment of the participant’s job-related needs. This should include the following, where appropriate (evidence of this should be found in the*  **Occupational Therapy ESSVR Assessment Form** and/or the **Therapy Case Notes***):*

*Job analysis: This could include discussing the participant’s job in detail, taking note of their role and related tasks, relationships, history of work, pre-existing work issues (e.g., performance difficulties or personality clashes) and logistics around getting to and from work (e.g., driving, walking, public transport). This could also include a worksite visit. There should be some consideration about what work-related tasks/skills might be affected by the stroke.*

Select ‘Yes’ if ESSVR OT assessed the impact of the stroke on the participant’s job and extract evidence verbatim from case file.

Select ‘No’ if ESSVR OT did not assess the impact of the stroke on the participant’s job and detail any reasons for why this was not completed.

Select ‘Not Deliverable’ if the participant did not have a job prior to their stroke. Detail the reason.

## 1.4 ESSVR OT assesses the impact of the stroke on the participant’s family (desirable)

If appropriate, the impact on the family should be assessed. This can include any emotional, logistical or financial impact on family members (e.g., if the participant was the sole earner, there may be other pressures that will facilitate/act as a barrier to RTW).

Select ‘Yes’ if ESSVR OT assesses the impact of the stroke on the participant’s family and extract evidence verbatim from case file.

Select ‘No’ if ESSVR OT does not assess the impact of the stroke on the participant’s family where the participant has mentioned family members and detail any reasons for why this was not completed.

Select ‘Not Deliverable’ if the participant does not report any family members. Detail the reason.

## 1.5 ESSVR OT helps the participant plan a return to work and prepares them to return to work

Work preparation and RTW planning:

*Once the job assessment has been completed, the ESSVR OT should use the analysis to inform the participant’s work preparation and return-to-work planning. Evidence of this would include:*

*Discussion of RTW options: The ESSVR OT should have a discussion with the participant about a RTW timeline and goals. Consideration should be given to potential facilitators and barriers to RTW. A RTW plan may be explicitly detailed in the* **Therapy Case Notes***.*

*Work preparation: As informed by the job analysis, the ESSVR OT should begin to prepare the participant to return to work. Evidence of this could include the following:*

***Getting to work****: If the participant used to drive to work, or if their ability to drive is essential for them to RTW, this might include helping the participant reinstate their driving status. If the participant will need to use public transport, practice of doing so may also be included as evidence. If the participant is unable to take public transport independently, consideration of other resources (e.g., Access to Work) may be included as evidence.*

***Building work tolerance****: Participants may require assistance in setting a structured work routine. ESSVR OTs may encourage the participant to begin to mimic the work routine/schedule and monitor and fatigue or other barriers/facilitators. Methods of building up stamina and tolerance may be considered (e.g., pacing).*

***Demands of the job role****: ESSVR OTs may review the physical demands of the job, as detailed in the job analysis, and begin to incorporate activities to build on skills to support those demands. Possible domains for these demands include:*

***Physical demands****: Manual dexterity, strength, coordination, long periods of standing or sitting, bending, lifting and working at height. Potential activities to address these demands might include beginning to take regular walks, going to the gym, DIY, gardening and resuming previous hobbies).*

***Cognitive demands:***

*Memory: Potential activities to address any memory difficulties may include use of a diary, notes, emails or phone reminder for remembering appointments or other important tasks.*

*Concentration: This might include detailing how long the participant might be able to cope at a computer or sustain attention during a specific task. Potential activities to address concentration difficulties might include devising a routine to test and monitor attention/concentration span.*

*Communication: Participants may face difficulties in verbal communication, reading/writing emails, understanding verbal instructions. Participants may also be affected by aphasia. Liaison with Speech and Language Therapy services may be required (see Item 5).*

***Executive functioning:***

*Organisation/Planning: This might include a participant’s ability to plan a project. Possible activities to address these demands might include creating job-related project timelines.*

*Time management: This might include a participant’s ability to set and maintain routines. Possible activities to address these demands might include a timed practice of getting to work.*

*Multi-tasking: Possible activities to address this might include asking the participant to take notes of the session or asking them to work on the computer while watching TV.*

*Problem solving: Possible activities to address this might include the participant creating a monthly budget system to manage their money whilst on reduced pay/benefits or solve a work-related problem.*

***Emotional/ behaviour demands:*** *Participants may experience mood swings, emotional lability, low mood, anxiety, loss of confidence, irritability, aggressive outburst and/or impulsivity/disinhibition. If the ESSVR OT feels the participant would benefit from further support to address these demands, there should be evidence of a referral to psychological/counselling services (see Item 5).*

***Social demands:*** *Participants may find social interaction difficult following their stroke. ESSVR OTs may encourage the participants to explore different social environments (e.g., coffee shop, pub, supermarket, family gatherings). They may also encourage the participant to maintain contact with friends and discuss strategies to address this.*

Select ‘Yes’ if ESSVR OT helps the participant plan a return to work and prepares them to return to work and extract evidence verbatim from the case notes.

Select ‘No’ if the ESSVR OT does not help the participant plan a return to work or does not help the participant prepare to return to work and detail any reasons for why this was not completed.

Select ‘Not Applicable’ if the participant no longer intends to return to work. The participant does not have to return to the same role or employer to make a return to work. Instances where the participant intends to return to a different role/employer will still require return to work plan and work prep. Provide detail verbatim from the file.

1.6 ESSVR OT communicates in writing with stakeholders regarding work status

If written consent is given for the ESSVR OT to contact the participant’s employer, which will be detailed in the **VR Consent Form**, they should contact the employer as soon as possible. Disclosure to the employer regarding the participant’s status should be discussed with the participant beforehand. A conversation should take place around assessing work skills/competencies and whether there is a person in the workplace who may be responsible for these assessments. There should be evidence of a conversation around a graded return to work. If there is evidence of the employer not wanting to speak about return to work until the participant is more likely to return to work, there may be evidence of the ESSVR OT arranging to speak to the employer again in a few weeks. There should be written evidence of these conversations which should be included in the **Therapy Case Notes** and should be sent as letters to the participant and the employer. There should also be written evidence of notifying the GP/consultant of any plans around returning to work.

Select ‘Yes’ if the ESSVR OT communicated with the key stakeholders they had consent to communicate with, where appropriate, regarding the participant’s work status and extract evidence verbatim from the case notes. Select the source of evidence.

Select ‘No’ if the ESSVR OT did not communicate with key stakeholders they had the participant’s consent to communicate with and detail any reasons for why this was not completed. Select the source of evidence.

Select ‘Not Applicable’ if the ESSVR OT did not have the participant’s consent to contact stakeholders. Provide detail verbatim from the file. Select the source of evidence.

1.7 Coordinates VR across relevant sectors

Coordinating the participant’s VR across all sectors may involve the ESSVR OT liaising with other services (neuropsychological, OT, physio, SALT) that may have administered relevant assessments. Evidence of coordinating VR across all sectors may include:

Liaising with SALT for support with participants experiencing aphasia, for working out the best format for written/verbal communication and for establishing areas of deficit which might affect communication in the workplace.

Referring the participant to psychology/counselling/IAPT services to support mood and behavioural difficulties.

Liaising with neuropsychology services to assess and address cognitive difficulties where appropriate.

Liaising with a physiotherapist to assess and address any physical difficulties that may impact work.

Liaising with an exercise provider to support the participant in achieving their physical goals in the gym.

General Practitioner: The ESSVR OTs are asked to provide written communication to the participant’s GP to keep them aware of any plans to return to work.

Employer: If appropriate, the OT will be the one to coordinate the VR with the participant’s employer. They will arrange any meetings or worksite visits with the employer that may be required and communicate the outcomes of these meetings in written format (a copy of this will be stored in the case file).

Select ‘Yes’ if the ESSVR OT coordinated the participant’s VR across relevant sectors and extract evidence verbatim from the case notes.

Select ‘No’ if the ESSVR OT did not coordinate the participant’s VR across the relevant sectors they had the participant’s consent to communicate with and detail any reasons for why this was not completed.

Select ‘Not Applicable’ if the participant did not give consent for the ESSVR OT to communicate with any relevant sectors.

# Stage Two: Graded Return to Work

2.1 Provides education and advice to the participant

The ESSVR OT is expected to provide information and advice to the participant, the participant’s employer and, if appropriate, the participant’s family regarding the difficulties and likely limitations (as determined by the job assessment and the work preparation). There should be evidence of written correspondence (email or letter) and a copy stored in the **Therapy Case Notes**. There might also be evidence of the ESSVR OT using the ‘Work Checklist’ as detailed by the ESSVR manual.

Select ‘Yes’ if the ESSVR OT provides education and advice to the participant and extract evidence verbatim from the case notes.

Select ‘No’ if the ESSVR OT does not provide needed education and advice to the participant and detail any reasons why this was not completed.

Select ‘Not Applicable’ if the participant did not have any questions, concerns etc that would require education or advice.

2.2 ESSVR OT provides emotional support to the participant (desirable)

Evidence of emotional support may include the ESSVR OT acknowledging and/or identifying contributing or maintaining factors of low mood, anxiety etc. and suggesting ways to mitigate the problem (including signposting or referral).

Select ‘Yes’ if the ESSVR OT provides emotional support to the participant and extract evidence verbatim from the case notes.

Select ‘No’ if the ESSVR OT does not provide needed emotional support to the participant and detail any reasons why this was not completed.

Select ‘Not Applicable’ if the participant did not appear to need emotional support.

## 2.3 Provides education and advice to the employer

The ESSVR OT is expected to provide information and advice to the participant, the participant’s employer and, if appropriate, the participant’s family regarding the difficulties and likely limitations (as determined by the job assessment and the work preparation). There should be evidence of written correspondence (email or letter) and a copy stored in the **Therapy Case Notes**. There might also be evidence of the ESSVR OT using the ‘Work Checklist’ as detailed by the ESSVR manual.

Select ‘Yes’ if the ESSVR OT provides education and advice to the employer and extract evidence verbatim from the case notes.

Select ‘No’ if the ESSVR OT does not provide needed education and advice to the employer and detail any reasons why this was not completed.

Select ‘Not Applicable’ if the participant did not give the ESSVR OT consent to contact their employer or if the employer did not have any questions, concerns etc that would require education or advice.

## 2.4 ESSVR OT provides emotional support to the employer (desirable)

Select ‘Yes’ if the ESSVR OT provides emotional support to the participant’s employer and extract evidence verbatim from the case notes.

Select ‘No’ if the ESSVR OT does not provide needed emotional support to the participant’s employer and detail any reasons why this was not completed.

Select ‘Not Applicable’ if the participant did not give consent for the ESSVR OT to contact their employer or the participant’s employer did not appear to need emotional support.

## 2.5 Provides education and advice to the participant’s family (desirable)

The ESSVR OT is expected to provide information and advice to the participant, the participant’s employer and, if appropriate, the participant’s family regarding the difficulties and likely limitations (as determined by the job assessment and the work preparation). There should be evidence of written correspondence (email or letter) and a copy stored in the **Therapy Case Notes**. There might also be evidence of the ESSVR OT using the ‘Work Checklist’ as detailed by the ESSVR manual.

Provides education and advice to the participant’s family (desirable)

Select ‘Yes’ if the ESSVR OT provides education and advice to the participant's family and extract evidence verbatim from the case notes.

Select ‘No’ if the ESSVR OT does not provide needed education and advice to the participant's family and detail any reasons why this was not completed.

Select ‘Not Applicable’ if the participant’s family did not give the ESSVR OT consent to contact or discuss the participant with their family or if the participant’s family did not have any questions, concerns etc that would require education or advice.

## 2.6 ESSVR OT provides emotional support to the participant’s family (desirable)

Select ‘Yes’ if the ESSVR OT provides emotional support to the participant’s family and extract evidence verbatim from the case notes.

Select ‘No’ if the ESSVR OT does not provide needed emotional support to the participant’s family and detail any reasons why this was not completed.

Select ‘Not Applicable’ if the participant did not give consent for the ESSVR OT to contact their family or the participant’s family did not appear to need emotional support.

- 1. ESSVR OT negotiates a phased return to work

In negotiating the phased return to work, the ESSVR OT may be able to create a time limit based on the participant’s needs, but some employers may have a time limit on graded return to work (e.g., 4-8 weeks). In some cases, this may be flexible. The ESSVR OT must negotiate a graded return to work plan that suits both the employer and the participant.

Select ‘Yes’ if the ESSVR OT negotiated a phased return to work and extract evidence verbatim from the case notes.

Select ‘No’ if the ESSVR OT did not negotiate a phased return to work where return to work was feasible and detail any reasons why this was not completed.

Select ‘Not Applicable’ if the participant did not give consent for the ESSVR OT to contact the employer, if return to work was not feasible or if the participant requested to self-manage their return to work.

- 1. ESSVR OT mediates workplace adjustments

Following the negotiation of a phased return to work, the ESSVR OT will also advocate for the participant in negotiating any workplace adaptations or adjustments to assist the participant in returning to work. This will include a worksite visit, if possible. Evidence of this will be written correspondence (email or letter) and a copy stored in the **Therapy Case Notes**.

The ESSVR OT, if permitted, should ideally conduct a worksite assessment which can include the following:

Assessing the participant whilst they are conducting their job role

A walk-through/talk-through through the job role

Monitoring the participant through their first day of the graded return to work plan.

There should be a discussion to agree the participant’s hours, duties and relevant supervision.

Select ‘Yes’ if the ESSVR OT mediated workplace adjustments and extract evidence verbatim from the case notes.

Select ‘No’ if the ESSVR OT did not mediate workplaces adjustments where adjustments were required and detail any reasons why this was not completed.

Select ‘Not Applicable’ if the participant did not give consent for the ESSVR OT to contact the employer, if return to work was not feasible, if the participant requested to self-manage their return to work or if no workplace adjustments were required.

- 1. ESSVR OT provides mechanism for feedback based on work performance:

There should be a discussion around a monitoring plan. This should include who is going to provide feedback as well as when feedback should be given and a plan for a situation where the participant might require an earlier review date (I.e., if they are struggling). There should be evidence of discussion around what points the participant will be assessed on.

Select ‘Yes’ if the ESSVR OT provided a mechanism for feedback based on work performance and extract evidence verbatim from the case notes.

Select ‘No’ if the ESSVR OT did not provide mechanism for feedback based on work performance where return to work was feasible and channels for feedback available and detail any reasons why this was not completed.

Select ‘Not Applicable’ if the participant did not give consent for the ESSVR OT to contact the employer, if return to work was not feasible, if the participant requested to self-manage their return to work or if there were no channels for feedback available.

2.10 ESSVR OT continuously monitors the participant’s return to work to ensure sustainability and job retention

The ESSVR OT should review the feedback from any assessment of the participant’s return to work whether the feedback is from the employer or the participant themselves. There might be evidence of review in a **Tailored Adjustments Agreement Form** or a **Work Review Letter** in the **Therapy Case Notes**. The ESSVR OT should address concerns that arise from the review and communicate with the employer and participant about ways to approach these concerns (e.g., if a participant is experiencing fatigue, the ESSVR OT should provide information about managing fatigue and support the participant with this). Monitoring should be apparent throughout the graded return to work, at least until the participant is working the maximum attainable hours and achieving their desired work-related goals. The ESSVR OT should continue to monitor the participant through their consolidation period of their graded return to work.

Select ‘Yes’ if the ESSVR OT continuously monitored the participant’s return to work to ensure sustainability and job retention and extract evidence verbatim from the case notes.

Select ‘No’ if the ESSVR OT did not continuously monitor the participant’s return to work to ensure sustainability and job retention where return to work was feasible and detail any reasons why this was not completed.

Select ‘Not Applicable’ if return to work was not feasible.

# Stage Three: Job Retention

3.1 ESSVR OT identifies issues that arise within the return-to-work process with relevant stakeholders

There should be evidence of ‘checking in’ with the participant and/or employer and documentation in the **Therapy Case Notes** if any further issues arise.

Select ‘Yes’ if the ESSVR OT identified issues that arose within the return-to-work process with all relevant stakeholders and extract evidence verbatim from the case notes.

Select ‘No’ if the ESSVR OT did not identify issues that arose within the return-to-work process with all relevant stakeholders where return to work was feasible and detail any reasons why this was not completed.

Select ‘Not Applicable’ if the participant did not give consent for the ESSVR OT to contact the employer or other stakeholders or if there were no apparent issues that arose.

3.2 ESSVR OT addresses the issues that arise within the return-to-work process with all stakeholders.

There should be evidence, where necessarily and appropriate, of the ESSVR OT attempting to address the issues that arise in the return-to-work process. It should be noted that the ESSVR OTs are not expected to address or ‘solve’ issues outside of their remit.

Select ‘Yes’ if the ESSVR OT addressed issues that arose within the return-to-work process with all relevant stakeholders and extract evidence verbatim from the case notes.

Select ‘No’ if the ESSVR OT did not address issues that arose within the return-to-work process with all relevant stakeholders where return to work was feasible and the issues were in their remit to address and detail any reasons why this was not completed.

Select ‘Not Applicable’ if the participant did not give consent for the ESSVR OT to contact the employer or other stakeholders or if there were no apparent issues that arose.

3.3 ESSVR OT explores alternative duties and/or job roles for the participant where current work could not be sustained/ was not feasible

If the participant is not able to return to their previous role, the ESSVR OT should explore if there are alternative duties or job roles. This might be evidenced in liaison with HR or the participant’s employer’s Occupational Health team. This will be documented in the **Therapy Case Notes** and the **Therapy Content Form**

Select ‘Yes’ if the ESSVR OT explored alternative duties and/or job roles for the participant where current work could not be sustained/ was not feasible and extract evidence verbatim from the case notes.

Select ‘No’ if the ESSVR OT did not explore alternative duties and/or job roles for the participant where current work could not be sustained/ was not feasible and detail any reasons why this was not completed.

Select ‘Not Applicable’ if the participant made a successful return to work in same job role with same duties.

3.4 ESSVR OT practices gradual, appropriate disengagement from the intervention with the participant

Once the ESSVR OT is satisfied that the participant has had a stable and sustainable return to work, there should be evidence of their role gradually resembling more of a monitoring role, addressing any problems that may arise. This should continue until the employer and participant feel that no further work visits or contact is required.

Select ‘Yes’ if the ESSVR OT practices gradual, appropriate disengagement from the intervention with the participant and extract evidence verbatim from the case notes.

Select ‘No’ if the ESSVR OT does not practice gradual, appropriate disengagement from the intervention with the participant, where disengagement from the intervention is feasible and detail any reasons why this was not completed.

Select ‘Not Applicable’ if gradual disengagement from the intervention is not feasible (I.e., in cases where the participant requires the intervention until the 1-year post-randomisation cuff-off point.

3.5 ESSVR OT discusses gradual, appropriate disengagement from the intervention with the participant’s employer

Ideally, the ESSVR OT will write a letter to the employer to detail the disengagement from the intervention to thank them and to encourage them to make contact in the future should any difficulties arise. A copy of the **Exit Letter to the Employer** might be filed in the **Therapy Case Notes**.

Select ‘Yes’ if the ESSVR OT practices gradual, appropriate disengagement from the intervention with the participant’s employer and extract evidence verbatim from the case notes.

Select ‘No’ if the ESSVR OT does not practice gradual, appropriate disengagement from the intervention with the participant’s employer, where disengagement from the intervention is feasible and detail any reasons why this was not completed.

Select ‘Not Applicable’ if gradual disengagement from the intervention is not feasible (I.e., in cases where the participant requires the intervention until the 1-year post-randomisation cuff-off point) or the participant has not given consent for the ESSVR OT to contact the employer.

# Stage Four: Discharge Process

4.1 ESSVR OT and participant agree an appropriate timepoint for withdrawing from the intervention

This involves agreeing the point at which the participant feels able to cope independently and no further intervention is needed from the vocational service. The discharge process must be agreed between the ESSVR OT and the participant’s family. This should be noted in the **Discharge Letter.**

Select ‘Yes’ if the ESSVR OT and participant agreed an appropriate timepoint for withdrawing from the intervention and extract evidence verbatim from the case notes.

Select ‘No’ if the ESSVR OT and participant did not agree an appropriate timepoint for withdrawing from the intervention where withdrawing from the intervention is feasible.

Select ‘Not Applicable’ if withdrawing from the intervention is not feasible or if participant becomes uncontactable.

## 4.2 Discusses and communicates the mechanism for re-accessing the vocational service or provides information about access to further avenues of support to the participant

The ESSVR OT must offer a re-accessible service to the participant, so long as the participant re-accesses the services within the 12-month post-randomisation window. The **Discharge Letter** will detail this fact as well as how the participant can re-access the service and a copy of this will be stored in the **Therapy Case Notes**.

Select ‘Yes’ if the ESSVR OT discusses and communicates the mechanism for re-accessing the vocational service or provides information about access to further avenues of support to the participant and extract evidence verbatim from the case notes.

Select ‘No’ if the ESSVR OT does not discuss and communicates the mechanism for re-accessing the vocational service or provides information about access to further avenues of support to the participant and detail any reasons why this was not completed.

There should not be a reason for this component to be undeliverable. Even in instances where a participant has disengaged from the intervention, the ESSVR OT should have communicated the mechanism for re-accessing the service in a letter to the participant.

## 4.3 Discusses and communicates the mechanism for re-accessing the vocational service or provides information about access to further avenues of support to the participant’s employer

Select ‘Yes’ if the ESSVR OT discusses and communicates the mechanism for re-accessing the vocational service or provides information about access to further avenues of support to the participant’s employer and extract evidence verbatim from the case notes.

Select ‘No’ if the ESSVR OT does not discuss and communicates the mechanism for re-accessing the vocational service or provides information about access to further avenues of support to the participant’s employer, where present, and detail any reasons why this was not completed.

Select ‘Not Applicable’ if the participant did not give the ESSVR OT consent to contact their employer or if the participant is self-employed.

4.4 ESSVR OT discusses and communicates the mechanism for re-accessing the vocational service or provides information about access to further avenues of support to the participant/ participant’s employer/ participant’s family (desirable)

Select ‘Yes’ if the ESSVR OT discusses and communicates the mechanism for re-accessing the vocational service or provides information about access to further avenues of support to the participant’s family and extract evidence verbatim from the case notes.

Select ‘No’ if the ESSVR OT does not discuss and communicates the mechanism for re-accessing the vocational service or provides information about access to further avenues of support to the participant’s family, where present, and detail any reasons why this was not completed.

Select ‘Not Applicable’ if the participant did not give the ESSVR OT consent to contact or discuss the participant with their family.

4.5 ESSVR OT provides the participant’s GP and other relevant health care professionals with a copy of the Discharge Letter

The **Discharge Letter** should be copied and sent to the participant’s GP and other healthcare professionals. This should be documented within the **Therapy Case Notes**.

Select ‘Yes’ if the ESSVR OT provided the participant’s GP and other relevant health care professionals with a copy of the Discharge Letter and extract evidence verbatim from the case notes.

Select ‘No’ if the ESSVR OT did not provide the participant’s GP and other relevant healthcare professionals with a copy of the Discharge Letter and detail any reasons why this was not completed.

Select ‘Not Applicable’ if the participant did not give consent for the ESSVR OT to contact their GP or other relevant healthcare professionals.

# Other Modifications to the ESSVR Protocol

Participant does not want the ESSVR OT to go into work:

1. *If the participant does not want the ESSVR OT to go into work due to concerns (e.g., manager support, attitudes of colleagues), the ESSVR OT must keep in contact with the participant and attempt their concerns over time. There should be evidence of monitoring this situation and addressing the workplace concerns with the participant in the* **Therapy Case Notes***.*
2. *If the participant is willing, the ESSVR OT should supply the participant’s employer with relevant resources (e.g., a guide written for employers about how to support their employee after stroke). There should be evidence of the ESSVR OT discussing this with the participant in the* **Therapy Case Notes***.*

The participant is self-employed, or their employer is no longer present:

1. *If the participant is self-employed, or the employer is uncontactable, the ESSVR OT must supply the participant with the same information that would have been given to the employer.*
2. *There should be evidence of the ESSVR OT attempting to create a graded return to work plan with the participant. This evidence will be found in the* **Therapy Case Notes***.*
3. The ESSVR OT should also encourage the participant to find a means to receive feedback on their performance. Evidence of this should be found in the **Therapy Case Notes**.

# Fidelity Checklist Completion

1. Fill in Participant ID, located at the top of the spreadsheet. This will be made up of a site code followed by a participant-specific ID number.
2. Fill in Researcher Initials. This will help us keep track of who has scored which participant.
3. For all components, you will be asked if there is evidence of the component. To answer, select from the drop-down menu either ‘Yes’, ‘No’ or ‘Not Deliverable’
4. For all components, you will be asked which data source you have extracted from. Select from the drop-down list. If you have extracted from multiple sources, please make a note where possible.
5. For component 1.1, input the date of the participant’s stroke into the spreadsheet. An automatic formula will calculate the date that the ESSVR OT must have made first contact by. Input the date of first contact (format: DD/MM/YYYY) and list any moderating factors in the appropriate box if the OT did not manage to contact the participant within 12 weeks.
6. A formula has been calculated to automatically take the fidelity scores from the ‘Yes/No/Not Deliverable’ boxes and tally the scores overall and per each individual stage. Please double check to make sure there are no errors.
7. At the top of the spreadsheet, please estimate the amount of time it took you to complete the checklist to the nearest quarter of an hour and enter it into the box titled ‘Time Taken to Complete’.
